# Mapping of Cognitive Pathways in Indian Ageing Population

**DOI:** 10.1002/alz.088160

**Published:** 2025-01-03

**Authors:** Suman Kushwaha, Puneet Talwar, Rachna Agarwal, Vibha Sharma, Aldrin Anthony, Rajinder Dhamija

**Affiliations:** ^1^ IHBAS, New Delhi, New Delhi, Delhi India; ^2^ University of Leigh, Belgium Belgium; ^3^ Institute of Human Behaviour and Allied Sciences, Delhi India; ^4^ Instutute of Human Behaviour and Allied Sciences, Delhi, Delhi India; ^5^ Institute of Human Behaviour and Allied Sciences, Delhi, Delhi India

## Abstract

**Background:**

Cognitive Reserve(CR) a concept based on the brain plasticity, is a mechanism that delays or minimizes clinical manifestations of brain changes due to aging. Prospective epidemiologic studies non‐demented individuals have shown that education, occupational duration and complexity, and greater lifetime engagement in cognitively stimulating activities are associated with a reduced risk of dementia. We study the cognitive reserve and its neuroimaging correlate.

**Methods:**

Prospective cross‐sectional study. 500 subjects were screened, Recruitment of subjects with inclusion criteria of age 40‐80 yr without neurodegenerative or psychiatric disorder. Clinical History and presence of CVD risk factors were investigated. MMSE, MOCA, Selected neuropsychology tests were done to evaluate cognitive status, CR assessed by CRIq scale. Biochemical and MRI(volumetry) and DTI(FA, Diffusivity.

**Result:**

252 pts were recruited, Mean age ‐53 yrs. 33% female. Diabetes(17%) and hypertension(24%). Mean MOCA score 24.CRIq was high in 10 subjects and majority had medium score. Significant association was found between Verbal and Category fluency with low CRIq and Serum ferritin with brain volume. Fractional isotropy(FA) values were reduced in central part of corpous callosum corelated with verbal fluency.

**Conclusion:**

Cognitive reserve can play a significant modifiable risk factor for cognitive decline. Neuroimaging can play an important biomarker to diagnose and in prognosis of cognitive impairment.

